# The Class I Scavenger Receptors CD5 and CD6 Play a Role in the Early Peritoneal Immune Response to *Echinococcus granulosus* Tegumental Antigens

**DOI:** 10.3390/ijms27062870

**Published:** 2026-03-22

**Authors:** Joaquín García-Luna, Cristina Català, Sylvia Dematteis, Francisco Lozano, María Velasco-De-Andrés, Gustavo Mourglia-Ettlin

**Affiliations:** 1 Área Inmunología, Departamento de Biociencias (DEPBIO), Facultad de Química, Universidad de la República, Montevideo 11600, Uruguay; jgarcialuna@fcien.edu.uy (J.G.-L.); sylvia.dematteis@gmail.com (S.D.); 2 Unidad Asociada de Inmunología, Instituto de Química Biológica (IQB), Facultad de Ciencias, Universidad de la República, Montevideo 11600, Uruguay; 3 Departamento de Inmunología, Instituto de Higiene “Prof. Arnoldo Berta”, Universidad de la República, Montevideo 11600, Uruguay; 4 Graduate Program in Chemistry, Facultad de Química, Universidad de la República, Montevideo 11600, Uruguay; 5 Immunoreceptors del Sistema Innat i Adaptatiu, Institut d’Investigacions Biomèdiques August Pi i Sunyer (IDIBAPS), 08036 Barcelona, Spain; cristina.catala7@gmail.com (C.C.); flozano@clinic.ub.es (F.L.); 6 Servei d’Immunologia, Hospital Clínic de Barcelona, 08036 Barcelona, Spain; 7 Departament de Biomedicina, Facultat de Medicina, Universitat de Barcelona, 08036 Barcelona, Spain

**Keywords:** *Echinococcus granulosus*, CD5, CD6

## Abstract

Scavenger Receptors (SRs) comprise a structurally diverse group of pattern recognition receptors (PRRs) involved in sensing non-self (microbial-associated molecular patterns) or altered-self ligands. CD5 and CD6 are class I SRs (SR-I) preferentially expressed by lymphoid cells and characterized by the presence of several tandem scavenger receptor cysteine-rich (SRCR) domain repeats. Both receptors interact with diverse microbial structures, including tegumental antigens from *Echinococcus granulosus* sensu lato (s.l.), the cestode parasite responsible for cystic echinococcosis (CE). This is notable as very few PRRs are currently known to detect parasitic helminths and because the infusion of recombinant soluble CD5 and CD6 proteins has shown prophylactic effects in murine secondary CE. Herein, the role of CD5 and CD6 in early immune responses to *E. granulosus* s.l. tegumental antigens (PSEx) was analyzed using CD5 (*Cd5*^−/−^) and CD6 (*Cd6*^−/−^)-deficient mice. Peritoneal B cells and macrophages from wild-type mice displayed specific and dose-dependent PSEx binding, which was impaired in those from *Cd5*^−/−^ and *Cd6*^−/−^ mice, supporting direct and/or indirect roles in parasite recognition. Additionally, in vivo exposure of peritoneal exudate cells (PECs) from *Cd5*^−/−^ and *Cd6*^−/−^ mice to PSEx showed altered activation profiles, including changes in CD80/CD86 expression, impaired early production of natural polyreactive antibodies, and cytokine shift from a Th1/Th17 to a Th2 profile. These findings strongly support the involvement of CD5 and CD6 receptors in the early immune recognition of *E. granulosus* s.l. antigens by PECs and influence immune responses critical for host resistance, highlighting their relevance in host–parasite interactions.

## 1. Introduction

The ability of the mammalian innate immune system to recognize pathogens relies on a limited number of germline-encoded receptors, named pattern recognition receptors (PRRs), which have evolved to identify conserved and essential microbial structures—not shared by the host—collectively known as microbial-associated molecular patterns (MAMPs) [1]. Among PRRs, Scavenger Receptors (SRs) comprise a large group of structurally diverse membrane-bound and/or soluble protein receptors that participate in a wide range of biological functions following the binding to multiple non-self or altered-self ligands [2]. Currently, two SR groups (namely SR-A and SR-I) are characterized by the presence of one or multiple repeats of an ancient and highly conserved cysteine-rich protein domain known as SRCR (for scavenger receptor cysteine-rich) [3].

Although a common single unifying function has not yet been reported for SRs, an increasing number was reported to interact with a broad range of MAMPs [4]. This is the case for the functionally and structurally highly homologous lymphocyte SR-I receptors CD5 and CD6, which are both mainly expressed on all T cells and B1a cells [3]. The ectodomains of both receptors are exclusively composed of three consecutive SRCR domains showing extensive sequence identity [5]. Their diverging cytoplasmic tails are devoid of intrinsic catalytic activity, but both contain phosphorylable motifs compatible with signaling engagement [3]. CD5 and CD6 are physically associated with the clonotypic antigen-specific receptor complex present on T and B1a cells (TCR and BCR, respectively) [6,7], and are involved in the fine-tuning of their transduced signals [8]. Interestingly, CD5 and CD6 also exhibit PRR activity. In this regard, while CD6 was shown to recognize bacterial LPS and LTA [9,10], CD5 was reported to bind diverse fungi species through recognition of β-glucans [11]. Additionally, both receptors not only were reported to interact with human viruses [12,13], but also were shown to recognize and bind tegumental antigens from the helminth *Echinococcus granulosus* sensu lato (s.l.) [14]. Thus, CD5 and CD6 might exhibit broad PRR activity during the early detection of diverse groups of pathogens.

Cystic echinococcosis (CE) is a cosmopolitan and globally distributed zoonosis caused by the larval stage of *E. granulosus* s.l., a genetically diverse complex of cestode parasites [15]. Primary CE occurs in intermediate hosts (domestic and wild ungulates; accidentally humans) after the ingestion of oncosphere-containing eggs, which later develop into metacestodes—or hydatid cysts—mainly in the liver and/or lungs of the infected host. Secondary CE occurs after the accidental spillage of protoscoleces (PSC) contained within primary fertile metacestodes due to their remarkable developmental plasticity, which make PSC able to develop either into metacestodes when seeded within intermediate hosts or into adult worms if ingested by a definitive host (usually dogs) [16,17]. In humans, secondary CE is an important medical problem associated with the surgical removal of primary cysts, and, although actual percentages of secondary CE cases post-surgery are debatable, a recent metanalysis reported global rates up to 17% [18].

In most helminthiases, including CE, parasite-induced immune responses are often associated with polarized and stereotyped Th2-type responses that are not necessarily associated with protective immunity [19,20,21]. Therefore, the role of innate receptors able to respond to helminth-derived components is highly relevant, and the fact that CD5 and CD6 recognize tegumental antigens from *E. granulosus* s.l. is noteworthy since few PRRs have currently been shown to detect pathogenic helminths [22,23]. We have previously reported the prophylactic effects of soluble recombinant human CD5 or CD6 (rshCD5 and rshCD6) protein infusions in the widely used mouse model of secondary CE [24]. Indeed, rshCD5 or rshCD6-treated mice showed significant reductions in the proportion of infected mice, as well as in the number of developed hydatid cysts and total parasite load [14]. Interestingly, tegumental antigens from *E. granulosus* s.l. reported to bind rshCD5/rshCD6 were further identified as highly relevant for the parasite’s survival [25], while the prophylactic effects of rshCD5/rshCD6 were shown to depend on both intrinsic antiparasitic activity on PSC as well as immunomodulatory effects during early murine secondary CE [26]. Herein, the involvement of membrane-bound CD5 and CD6 in the early peritoneal immune response induced by tegumental antigens from *E. granulosus* s.l. was analyzed using knockout mice.

## 2. Results

### 2.1. Parasite Tegumental Antigens Bind to Peritoneal B Cells and Macrophages

**Figure 1:**
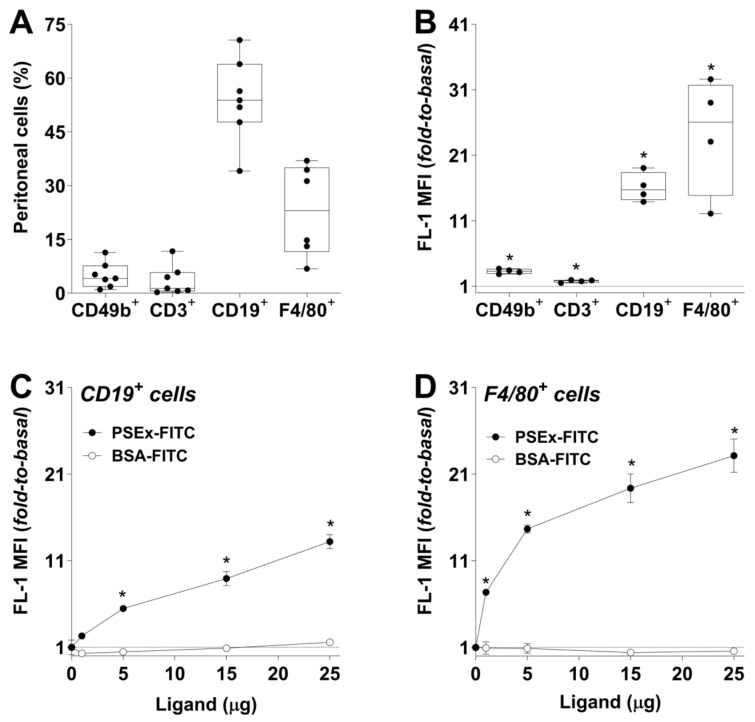
**Parasite tegumental antigens bind to peritoneal B cells and macrophages.** In vivo analysis of PSEx interaction with NK (CD49b^+^), T (CD3^+^), B cells (CD19^+^) and macrophage (F4/80^+^) cells was determined by flow cytometry in PEC isolated from naïve C57Bl/6 mice at 30 min *p.i.* of PSEx-FITC (100 μg) or PBS (*i.p.*). Results are represented as total population percentage (**A**) and as the ratio (fold-to-basal) of MFI values (FL-1) from PSEx-FITC- vs. PBS-treated mice for each cellular population (basal = inoculation of PBS) (**B**). In vitro incubation (30 min at 4 °C) of increasing amounts (1, 5, 15 or 25 μg) of PSEx-FITC/BSA-FITC with PEC from naïve C57Bl/6 mice labeled with CD19-specific (**C**) or F4/80-specific (**D**) fluorochrome-conjugated antibodies. Results are reported as fold-to-basal values (basal = absence of FITC-labeled probes). Parametric and non-parametric data were compared using one-way ANOVA and Mann–Whitney–Wilcoxon test, respectively (*, *p* < 0.05). Results in (**A**,**B**) are depicted as box-and-whiskers, while those in (**C**,**D**) are shown as mean ± SEM values.

PSEx is a soluble antigenic fraction enriched in tegumental antigens from viable PSC of *E. granulosus* s.l., which has been shown to contain relevant immunomodulatory and protective antigens [27,28,29,30]. Additionally, it has been proved useful for mimicking the early immune response induced during murine secondary CE [31]. Thus, the physical interaction between tegumental antigens from *E. granulosus* s.l. and PEC from WT C57Bl/6 mice was first analyzed in vivo. To that end, mice were *i.p.* infused with FITC-conjugated PSEx (PSEx-FITC), as well as with PBS used as basal fluorescence control. PEC samples were recovered 30 min *p.i.* and separately stained with lineage-specific PE-conjugated antibodies for subsequent flow cytometry analyses (Figure S1). Peritoneal cell-lineages analyzed were NK (CD49b^+^), T (CD3^+^), B (CD19^+^) and macrophage (F4/80^+^) cells, which together accounted for approximately 90% of total PEC, being B cells and macrophages roughly 50% and 30% of total PEC, respectively, as expected (Figure 1A).

In order to assess the physical interaction of PSEx-FITC with PEC, and to avoid potential cell type-specific autofluorescence phenomena, we further determined the ratio (fold-to-basal) of MFI values (FL-1) for each cell population. Results showed that, although all PEC populations analyzed were capable of binding PSEx-FITC, B cells (CD19^+^) and macrophages (F4/80^+^) exhibited higher MFI values, with approximate fold-to-basal values of 20 and 30, respectively (Figure 1B).

Given the highest PSEx binding capacity of peritoneal B cells and macrophages observed in vivo, further in vitro studies were conducted to characterize these interactions. Thus, PEC from naïve C57Bl/6 mice were incubated with increasing amounts (1, 5, 15 or 25 μg) of PSEx-FITC, or equivalent amounts of BSA-FITC as a specificity control, and then, labeled with PE-conjugated anti-CD19 or anti-F4/80 antibodies for flow cytometry analyses (Figure S2). Again, results were reported for each cellular population as fold-to-basal values between PEC incubated in the presence or absence of FITC-labeled probes. The results showed that PSEx-FITC binding to peritoneal B cells (CD19^+^) and macrophages (F4/80^+^) was specific and dose-dependent. Notably, B cells exhibited lower binding avidity than macrophages: while B cells reached a roughly 6-fold significant increase over basal levels with 5 µg of PSEx-FITC (Figure 1C), macrophages required only 1 µg to achieve similar results (Figure 1D).

### 2.2. Absence of CD5 and CD6 Expression Impairs Binding of Tegumental Antigens to B Cells and Macrophages

Knockout mouse strains constitute useful tools to dissect the involvement of particular molecules in complex biological phenomena, like the immune response to parasite antigens. Thus, the relevance of CD5 and CD6 receptors during the early peritoneal immune response induced by tegumental antigens from *E. granulosus* s.l. was evaluated using *Cd5*^−/−^ and *Cd6*^−/−^ mice as biological models [32,33]. Following the above-described strategy, we first evaluated the effect(s) of deficient CD5 or CD6 expression in the physical interaction of tegumental antigens with peritoneal B cells and macrophages. To that end, PEC from naïve *Cd5*^−/−^ and *Cd6*^−/−^ mice as well from their corresponding WT littermates were incubated with increasing amounts of PSEx-FITC, and then, labeled with anti-CD19 or anti-F4/80 PE-conjugated antibodies for flow cytometry analyses (Figure S2). For each cellular population, results were reported as fold-to-basal values between PEC incubated in the presence vs. absence of PSEx-FITC.

**Figure 2:**
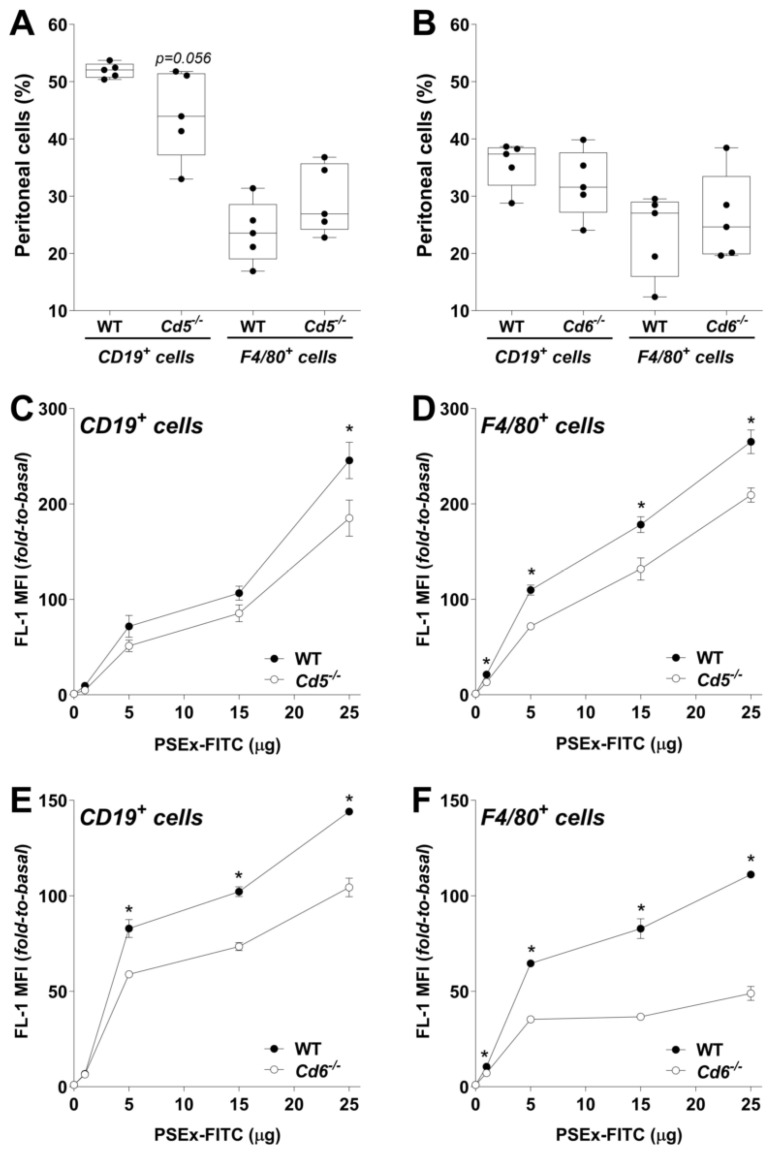
**Absence of CD5 and CD6 expression impairs binding of tegumental antigens to B cells and macrophages.** PEC from naïve *Cd5*^−/−^ and *Cd6*^−/−^ mice (and corresponding WT littermates were incubated for 30 min at 4 °C with increasing amounts (1, 5, 15 or 25 μg) of PSEx-FITC, and then, stained with anti-CD19 (B cells) or anti-F4/80 (macrophages) PE-conjugated antibodies. Flow cytometry results were reported as fold-to-basal values (basal = absence of FITC-PSEx). Compared composition of peritoneal B cells and macrophages in *Cd5*^−/−^ and WT mice (**A**) or in *Cd6*^−/−^ and WT mice (**B**) are shown in percentages. PSEx binding to PEC from naïve *Cd5*^−/−^/WT mice incubated in vitro with increasing amounts of PSEx-FITC, and subsequently labeled with PE-conjugated antibodies for CD19 (**C**) or F4/80 (**D**). Results were reported also as fold-to-basal values (basal = absence of PSEx-FITC probe). Analogous studies performed for CD19^+^ cells (**E**) and F4/80^+^ cells (**F**) from *Cd6*^−/−^ and WT mice are shown. Parametric and non-parametric data were compared using Student’s *t*-test and Mann–Whitney–Wilcoxon test, respectively (*, *p* < 0.05). Results in (**A**,**B**) are depicted as box-and-whiskers, while those in (**C**–**F**) are shown as mean ± SEM values.

Potential differences in the peritoneal composition of B cells (CD19^+^) and macrophages (F4/80^+^) between KO and WT mice were first assessed. No significant differences were observed in *Cd5*^−/−^ nor in *Cd6*^−/−^ mice (Figure 2A,B) compared to their corresponding WT littermates, although a trend (*p* = 0.056) towards reduction in peritoneal B cells was observed in *Cd5*^−/−^ mice (Figure 2A). Median group values for CD19^+^ cells were 52% and 44% in WT and *Cd5*^−/−^ mice, respectively, while the values for WT and *Cd6*^−/−^ mice were 37% and 32%, respectively (Figure 2A,B). Regarding peritoneal macrophages, median group values for F4/80^+^ cells were 24% and 27% in WT and *Cd5*^−/−^ mice, respectively, while these values were 27% and 25% in WT and *Cd6*^−/−^ mice, respectively (Figure 2A,B).

Next, PSEx binding to peritoneal B cells and macrophages from KO and WT mice was assessed as described above. Overall, deficient CD5 or CD6 expression impaired PSEx binding in both cell types, with the extent of the effect varying depending on the receptor and cell type. Regarding *Cd5*^−/−^ mice, peritoneal B cells were less affected than macrophages in their PSEx binding ability (Figure 2C,D). Indeed, CD19^+^ cells from *Cd5*^−/−^ mice exhibited a slight but significant decrease in PSEx binding only at the highest antigen concentration, while F4/80^+^ cells exhibited impaired binding across all tested concentrations (Figure 2C,D). Similarly, B cells from *Cd6*^−/−^ mice were less affected than macrophages in their PSEx binding ability (Figure 2E,F), although to a lesser extent than B cells from *Cd5*^−/−^ mice (Figure 2C). Unlike *Cd5*^−/−^ B cells, CD19^+^ cells from *Cd6*^−/−^ mice displayed significant binding reductions starting at 5 µg of PSEx-FITC (Figure 2E). F4/80^+^ cells from *Cd6*^−/−^ mice showed similar but more pronounced impairments compared to those from *Cd5*^−/−^ mice (Figure 2F).

### 2.3. Effects of CD5 and CD6 Expression on the Activation of B Cells and Macrophages Induced by Tegumental Antigens

Since peritoneal B cells and macrophages, the two major PEC populations, were shown to specifically interact with tegumental antigens, we further explored the early peritoneal immune response in PSEx-treated *Cd5*^−/−^ and *Cd6*^−/−^ mice. To that end, *Cd5*^−/−^ and *Cd6*^−/−^ mice, and their corresponding WT littermates, were *i.p.* infused with 50 μg of PSEx or sterile PBS, and 48 h *p.i.* PEC and cell-free peritoneal exudates were obtained. Flow cytometry studies were first performed to evaluate the activation status of peritoneal B cells and macrophages following PSEx infusion; and since both populations are professional antigen presenting cells, the surface expression of co-stimulatory molecules (CD80 and CD86) was analyzed.

**Figure 3:**
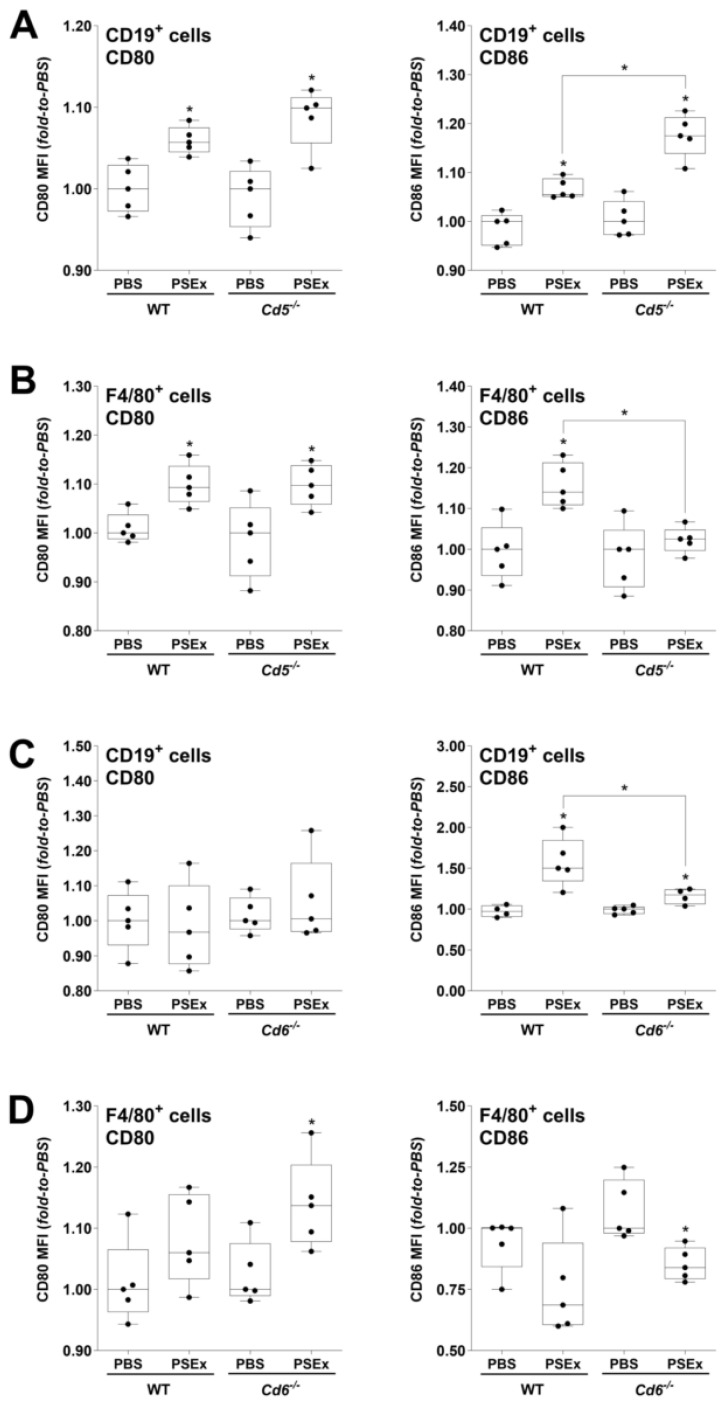
**Effects of CD5 and CD6 expression on the activation of B cells and macrophages induced by tegumental antigens.** PEC were isolated from *Cd5*^−/−^ and *Cd6*^−/−^ mice and corresponding WT littermates at 48 h *p.i.* of PSEx (50 μg) or PBS infusion. Assessment of CD80 and CD86 expression by flow cytometry was performed on peritoneal B cells (**A**) and macrophages (**B**) from *Cd5*^−/−^ and WT mice, and on peritoneal B cells (**C**) and macrophages (**D**) from *Cd6*^−/−^ and WT mice. Results were reported as fold-to-basal of MFI values (basal = PBS inoculation). Non-parametric data were compared using Mann–Whitney–Wilcoxon test (*, *p* < 0.05). Results are depicted as box-and-whiskers.

Regarding *Cd5*^−/−^ mice, similar CD80 expression patterns to their WT littermates were observed in peritoneal B cells and macrophages (Figure 3A,B) after PSEx infusion. However, the CD86 expression in CD19^+^ cells was significantly higher in *Cd5*^−/−^ vs. WT cells following PSEx infusion (Figure 3A), while absence of induction was observed in *Cd5*^−/−^ F4/80^+^ cells (Figure 3B). Contrary to *Cd5*^−/−^ mice, *Cd6*^−/−^ ones displayed less pronounced effects on the activation status of peritoneal B cells and macrophages (Figure 3C,D). Indeed, only CD86 expression in peritoneal B cells showed a significant difference between PSEx-treated WT vs. *Cd6*^−/−^ mice, with a less intense induction in the last group (Figure 3C).

### 2.4. Effects of CD5 and CD6 Expression on the Production of Natural Polyreactive Antibodies Induced by Tegumental Antigens

The functional consequences of deficient CD5 and CD6 expression on the early B cell immune response to tegumental antigens were evaluated in terms of local production of natural polyreactive antibodies (NAbs). To that end, the level of IgM, IgA and IgG subclasses specific to DNP—a surrogate of natural polyreactivity—were analyzed by ELISA in the peritoneal cell-free exudates. Samples were collected as previously described following PSEx infusion in *Cd5*^−/−^ and *Cd6*^−/−^ mice, and in their corresponding WT littermates.

**Figure 4:**
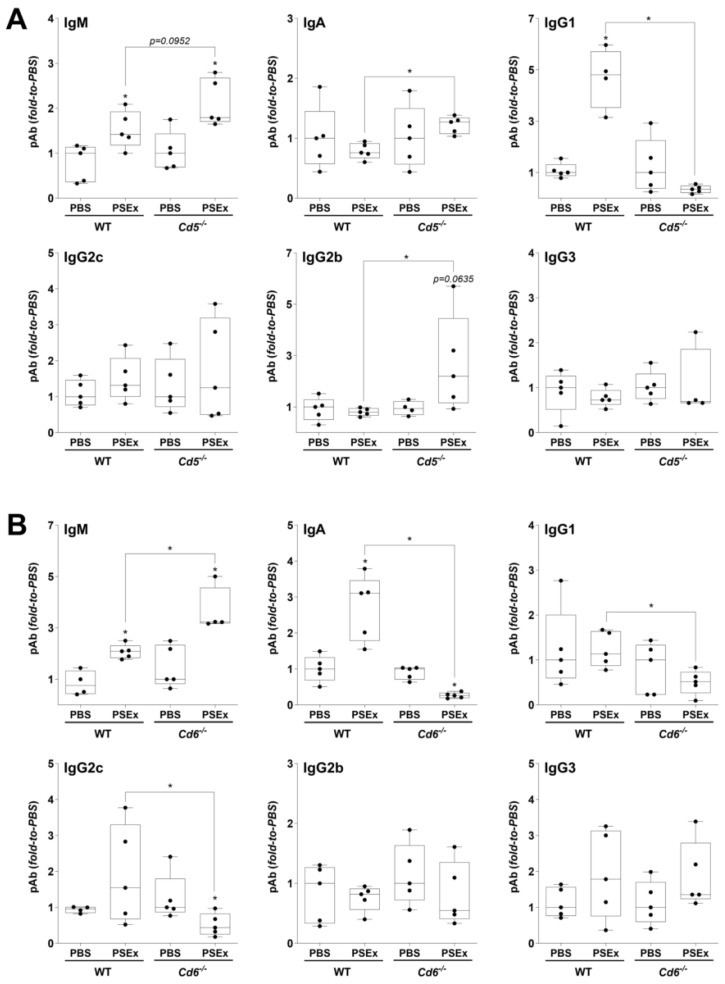
**Effects of CD5 and CD6 expression on the production of NAb induced by tegumental antigens.** Levels of IgM, IgA and IgG subclasses specific for DNP—a surrogate of polyreactivity—were analyzed by ELISA in peritoneal cell-free exudates obtained 48 h after *i.p.* inoculation of PSEx (50 μg) or PBS in *Cd5*^−/−^ (**A**) and *Cd6*^−/−^ (**B**) mice, and their corresponding WT littermates. NAb levels were normalized to the corresponding PBS-inoculated controls (either from KO or WT mice). Non-parametric data were compared using Mann–Whitney–Wilcoxon test (*, *p* < 0.05). Results are depicted as box-and-whiskers.

Regarding CD5 expression, NAb IgA levels showed a significant increase in PSEx-treated *Cd5*^−/−^ vs. WT mice, along with a similar tendency in IgM (*p* = 0.0952) and IgG2b (*p* = 0.0635) levels. Inverse results were observed for NAb IgG1 levels (Figure 4A). On the other hand, more notable results regarding CD6 expression were observed for NAb IgM, IgA and IgG2c. Similarly to *Cd5*^−/−^ mice, NAb IgM levels showed a significant increase in PSEx-treated *Cd6*^−/−^ vs. WT mice together with a significant decrease in the NAb IgG1 levels. However, opposite results were obtained for NAb IgA and IgG2c, since the increases in their levels after PSEx exposure in *Cd6*^−/−^ mice reverted to suppressions in PBS-treated *Cd6*^−/−^ mice (Figure 4B).

### 2.5. Effects of CD5 and CD6 Expression on the Cytokine Response Induced by Tegumental Antigens

Finally, the impact of deficient CD5 and CD6 expression on the peritoneal cytokine response following PSEx infusion was further evaluated. To that end, cytokine expression profiles were analyzed by qRT-PCR in PEC from *Cd5*^−/−^ and *Cd6*^−/−^ mice, as well as in their corresponding WT littermates, following PSEx infusion as previously described.

**Figure 5:**
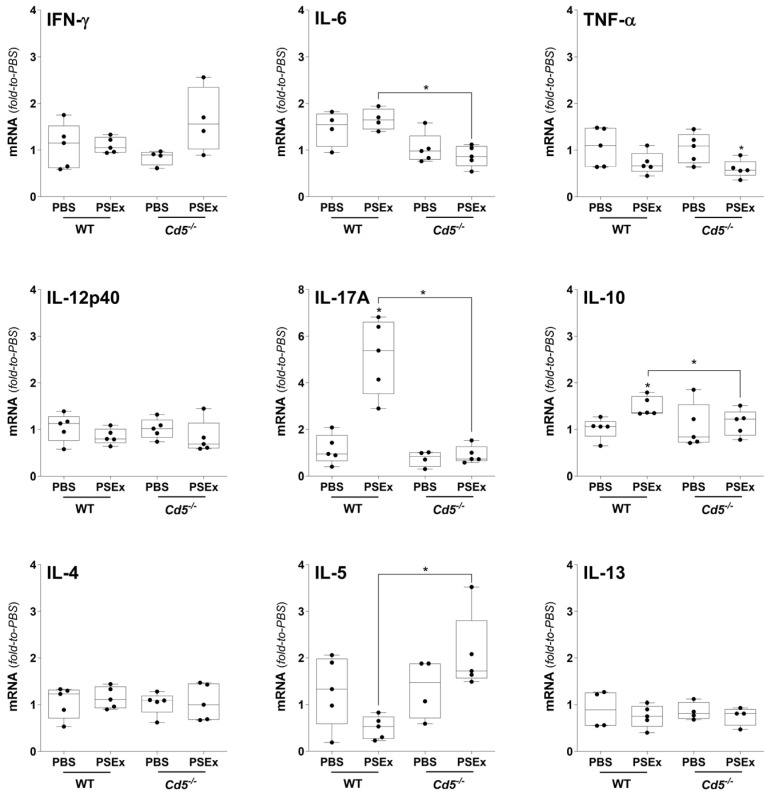
**Effects of CD5 expression on the cytokine response induced by tegumental antigens.** PEC from *Cd5*^−/−^ mice, and their corresponding WT littermates, were obtained 48 h *p.i.* of PSEx (50 μg) or PBS, and cytokine responses were evaluated through qRT-PCR. Levels of cytokine transcripts were normalized to the corresponding PBS-treated controls (either from KO or WT mice), and β-actin was used as the housekeeping gene. Non-parametric data were compared using Mann–Whitney–Wilcoxon test (*, *p* < 0.05). Results are depicted as box-and-whiskers.

Regarding *Cd5*^−/−^ mice, no significant differences were observed for IFN-γ, TNF-α, IL-12-p40, IL-4 and IL-13 cytokines following PSEx infusion with regard to WT mice (Figure 5). Differences were only observed for IL-6, IL-17A, IL-5 and IL-10. Thus, while IL-6, IL-17A and IL-10 expression were not upregulated in *Cd5*^−/−^ vs. WT mice following PSEx exposure, that of IL-5 was significantly upregulated (Figure 5).

**Figure 6:**
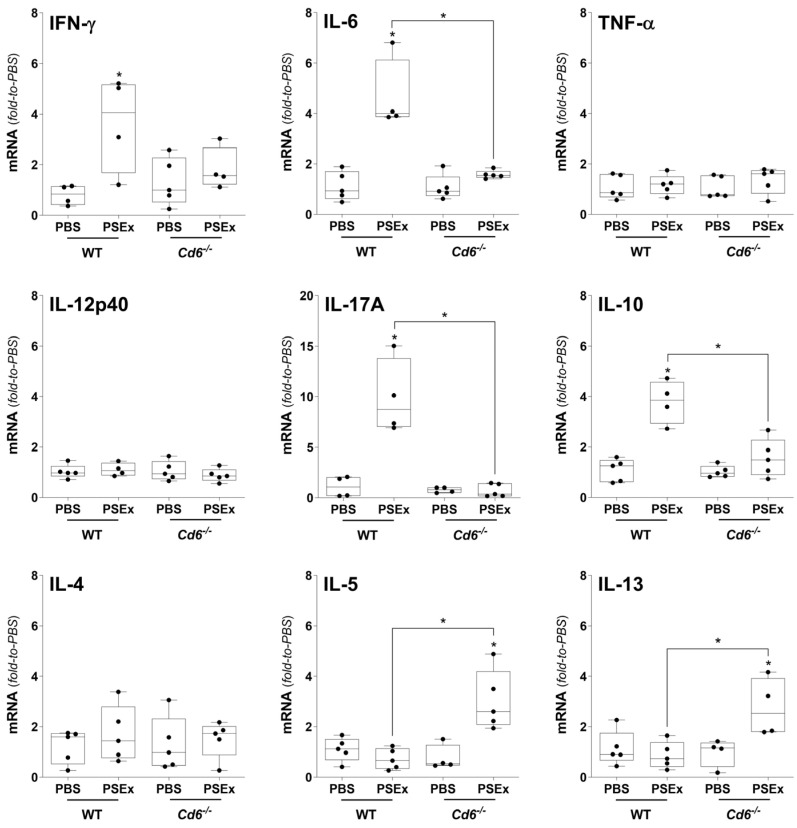
**Effects of CD6 expression on the cytokine response induced by tegumental antigens.** PEC from *Cd6*^−/−^ mice, and their corresponding WT littermates, were obtained 48 h *p.i.* of PSEx (50 μg) or PBS, and cytokine responses were evaluated through qRT-PCR. Levels of cytokine transcripts were normalized to the corresponding PBS-treated controls (either from KO or WT mice), and β-actin was used as the housekeeping gene. Non-parametric data were compared using Mann–Whitney–Wilcoxon test (*, *p* < 0.05). Results are depicted as box-and-whiskers.

A similar situation was observed for *Cd6*^−/−^ vs. WT mice. Again, PSEx exposure did not upregulate IL-6, IL-17A and IL-10 expression, while that of IL-5 and IL-13 was significantly upregulated (Figure 6).

## 3. Discussion

Previous reports from our group demonstrated direct binding of ectodomains from the SR-I CD5 and CD6 lymphocyte receptors to viable PSC and their tegumental antigens (PSEx) [14]. Furthermore, the *i.p.* administration of soluble CD5 and, to a lesser extent soluble CD6, exerted prophylactic effects in the murine model of secondary CE [14], either by direct antiparasite mechanisms and/or early immunomodulation [26]. Such effects were most likely derived from binding to relevant parasite tegumental interactors by soluble CD5 and CD6 [25]. These results are remarkable not only because very few innate receptors are currently known to interact with structural components from helminth parasites [34,35,36,37,38], but also because CD5 and CD6 receptors are both expressed in B1a cells, which are key players in helminthiases because of NAb and immunoregulatory cytokines (i.e., IL-10) production [39,40,41]. B1a cells are mostly located in the peritoneal cavity, where PSC develop into metacestodes [24], suggesting that cells expressing SR-I CD5 and CD6 receptors may play crucial roles in the early immune response to secondary CE.

B1a cells and macrophages are relevant representatives of peritoneal cellularity, as both cell subsets are characterized by the expression of multiple PRRs, including several receptors already reported to interact with helminth antigens. Among these are some members of the TLR family (TLR4, TLR3 and TLR2) [36,42,43,44,45], and of the SR class E group, such as Dectin-2 [46], CD206 [34,38,47], CLEC4F/CLECSF13 [35], and DC-SIGN/CD209a [37]. So, the first assessment of PEC’s ability from naïve WT mice to interact with parasite tegumental antigens (PSEx), showed that B cells and macrophages exhibited the greatest interaction capacity among PEC. Moreover, such ability was shown to be specific and dose-dependent for both populations (Figure 1). As previously stated, PSEx is a soluble fraction enriched in tegumental antigens from viable PSC of *E. granulosus* s.l., which has been described to contain relevant immunomodulatory as well as protective parasite components [27,28,29,30]. Moreover, PSEx inoculation has been proved useful for mimicking the early immune response induced during murine secondary CE [31]. At the molecular level, PSEx is a complex fraction composed of parasite proteins, carbohydrates and lipids [30]. Specifically, it contains more than 1,000 parasite proteins [30,31], some of them previously identified as receptor-specific and/or receptors-shared interactors for soluble CD5 and CD6 [31]. Thus, our results on PSEx binding to peritoneal B and macrophage cells suggested that some of those antigens, whether receptors-shared or -specific, might be involved in the observed physical interactions. Additional PEC types with high PSEx binding capacity (e.g., dendritic cells, mast cells, etc.) cannot be discarded since herein roughly 10% of PEC were not included among the analyzed cell populations (Figure 1A).

Next, the observed PSEx binding capacity to PEC was evaluated in cells from *Cd5*^−/−^ and *Cd6*^−/−^ mice. The results showed that PEC from *Cd5*^−/−^ and *Cd6*^−/−^ animals bound less PSEx-FITC than those from their WT littermates (Figure 2), thus indicating that CD5 and CD6 are also involved in such interactions. Accordingly, impairment of PSEx binding to peritoneal B cells from KO mice could be assigned—at least partially—to direct effects derived from deficient CD5/CD6 surface expression in the main peritoneal B cell subset (B1a cells). However, since the interaction was not fully abolished in cells from KO mice, other PRRs also expressed in peritoneal B cells might be involved. Unexpectedly, macrophages from both *Cd5*^−/−^ and *Cd6*^−/−^ mice showed an even greater reduction in PSEx binding, implying possible indirect but CD5/CD6-dependent mechanisms apart from the role of other PRRs. This potential effect(s) might derive from secondary alterations in the expression profile of diverse PRRs in macrophages due to improper physiological and/or basal communication with T cells [48,49]. Although currently less analyzed, these phenomena could also apply to the PSEx binding characteristics in peritoneal B cells from *Cd5*^−/−^ and *Cd6*^−/−^ mice. Additionally, the involvement of yet underestimated subsets of CD5^+^ and/or CD6^+^ peritoneal macrophages cannot be fully discarded, since minor subpopulations of CD5/CD6-expressing immune cells—other than T and B1a cells—have been less frequently described, i.e., NK cells [50] and dendritic cells [51,52,53,54,55]. The reported existence of biphenotypic B/macrophage cells (B220^+^ F4/80^+^) in mouse PEC should also be taken into consideration, as they result from conversion of CD5^+^ B1a cells into macrophages and are hypothesized to combat parasites that induce a Th2-type environment [56].

Peritoneal B cells and macrophages have been suggested to play relevant roles in murine secondary CE. Indeed, PSC from *E. granulosus* s.l. were shown to be susceptible to the killing by activated peritoneal macrophages [57,58], and associations between host resistance mechanisms and larger numbers of peritoneal macrophages have been reported [59]. Regarding peritoneal B cells, while PSC glycoconjugates were shown to induce the rapid secretion of both IL-10 and non-specific polyclonal antibodies [59], with both effects favoring the early development of inefficient antiparasite immune responses, they were also suggested to potentially produce NAb associated with host resistance to CE [40]. The specific recognition of tegumental antigens by membrane-bound SR-I CD5 and CD6 receptors may have relevant direct and indirect functional consequences on peritoneal B cells and macrophages. Both CD5 and CD6 are signal transducing receptors reported to induce MAPK-cascade activation following their binding to certain MAMPs [9,11]. Therefore, we further evaluated the influence of CD5/CD6 expression on the early peritoneal immune response triggered by the exposure to tegumental antigens. Regarding PSEx-induced activation of peritoneal B cells and macrophages, our results showed that CD5, and to a lesser extent CD6, played modulatory effects on the early expression of co-stimulatory molecules (Figure 3). Particularly, and unlike CD80 expression, significant differences were observed on the induction of CD86. However, since the resulting inhibition or induction of cell activation observed depends not only on the receptor evaluated but also on the cell type analyzed, it is quite risky to extract strong conclusions. Moreover, as diverse antigens from *E. granulosus* s.l. have been reported to induce non-canonical activation phenotypes in other antigen presenting cells (e.g., dendritic cells) [60,61], similar effects cannot be ruled out for tegumental antigens (PSEx) acting on peritoneal B cells and/or macrophages. Thus, whatever the receptor-specific effects (repression/induction), either direct and/or indirect, the SR-I CD5 and CD6 receptors seemed to play non-redundant roles in the early activation of such cell types in response to parasite tegumental antigens. However, further studies are warranted to assess the specific relevance of such modulatory effects in the overall antiparasite response.

Functional consequences derived from the alterations in the PSEx-induced B cell activation in *Cd5*^−/−^ and *Cd6*^−/−^ mice were indirectly evaluated through characterization of peritoneal antibody responses, since antibodies have been shown to be relevant during the immune response in various mouse models of parasitic infections [62,63,64,65,66,67,68]. Particularly, NAb were characterized by their usual polyreactivity [69,70], which gives them the ability to recognize a wide variety of antigens with low affinity [71,72]. In fact, NAb constitutes part of the first-line defense against pathogens, being able to delay their spread and/or increase their immunogenicity [69,73]. Regarding CE, our group previously suggested that IgG2b and IgG2c NAb partially mediated host protection in murine secondary CE [74], while particular profiles of NAb acted as interesting humoral biomarkers of host resistance to CE both in mice and in patients [40]. Thus, to get further insight into the role of CD5/CD6 on the early peritoneal B cell response induced by *E. granulosus* s.l. tegumental antigens, the influence of CD5 and CD6 expression on the peritoneal pAb profile induced by PSEx inoculation was assessed. The results showed the involvement of CD5 and CD6 receptors in modulating early peritoneal NAb production in response to tegumental antigens (Figure 4), with effects—either stimulatory or inhibitory—depending on both the receptor and the specific NAb isotype. The overall observed effects on NAb production might be derived, at least partially, from the observed effects of CD5/CD6 deficiency in the PSEx-induced peritoneal B cell activation (Figure 3), suggesting that receptors-dependent phenotype effects exert relevant functional consequences.

Besides their canonical effector functions, peritoneal B and macrophage cells are becoming recognized as key players in the early orchestration of the immune response to parasite antigens. Particularly, while peritoneal macrophages migrate to the omentum in response to different inflammatory stimuli to collaborate in the initiation (priming) and amplification of the adaptive response [75], B cells secrete cytokines that contribute to the differentiation of naïve CD4^+^ T cells [76,77]. Indeed, B cells are increasingly recognized as important actors during Th2-type immune responses against helminths, both participating in the production of protective antibodies as well as in controlling harmful inflammation and/or participating in regulatory responses [21]. Thus, the role of CD5/CD6 in the global peritoneal cytokine profile (including B cells, macrophages and other cell types) induced by *E. granulosus* s.l. tegumental antigens was finally characterized. In this regard, deficient CD5 or CD6 expression resulted in early modulatory effects on the PSEx-induced cytokine response, both in receptor- as well as in cytokine-dependent ways (Figure 5 and Figure 6). Remarkably, the PSEx-induced upregulation of IL-10 and IL-17A disappeared in the absence of either CD5 or CD6 expression, while IL-5 and IL-13 showed mostly inverse behaviors. Interestingly, these cytokines have been reported as relevant actors in CE. Accordingly, while the usually stereotyped helminth-induced Th2-type responses are associated with inefficient responses to combat *E. granulosus* s.l. infection, Th1/Th17-biased cytokine responses seemed to mediate host protection, both in murine models [59,74,78,79,80,81,82] and in patients [83,84,85,86,87,88]. Thus, while the administration of recombinant IL-17A has been reported to attenuate the growth of hydatid cysts and to reduce liver fibrosis in the murine model of secondary CE [82], the forced induction of IL-4 in infected mice led to higher cyst loads [80]. Therefore, the absence of CD5 or CD6 expression seemed to modulate the early PSEx-induced cytokine profile from an overall Th1/Th17-biased profile towards a more mixed cytokine response containing Th2-polarizing actors, thus potentially associating the expression of both receptors with host CE resistance mechanisms. Further studies are warranted to strictly conclude on this issue since cytokines were herein evaluated only at the mRNA level.

## 4. Materials and Methods

### 4.1. Animals

Animal experiments were performed in compliance with Comisión Honoraria de Experimentación Animal (CHEA, Universidad de la República, Uruguay), according to the National Uruguayan Legislation N°18.611. The study was conducted in accordance with the Declaration of Helsinki and approved by the Ethics Committee of Facultad de Química (Universidad de la República, Uruguay) (approval code and date: 101900-000361-16 on 2 June 2016). Female wild-type C57Bl/6 mice were obtained from DILAVE (Uruguay) and housed at the animal facility of Instituto de Higiene (Uruguay), while *Cd5*^−/−^ and *Cd6*^−/−^ female mice, and their corresponding wild-type littermates (C57Bl/6N for *Cd5*^−/−^ and C57Bl/6J for *Cd6*^−/−^) [32,33], were bred and housed at the animal facility of Facultat de Medicina—Universitat de Barcelona (Barcelona, Spain). Mice were housed under conventional conditions, and were fed ad libitum with sterilized food and water.

### 4.2. Parasites and Antigens

Parasites were obtained as previously reported [24]. Briefly, fertile bovine hydatid cysts from Uruguayan abattoirs were aseptically punctured to obtain PSC. Parasites were extensively washed with PBS pH 7.4 containing antibiotics (penicillin 60 μg/mL, streptomycin 100 μg/mL, and amphotericin-B 250 ng/mL), and viability was assessed by eosin exclusion staining. Only PSC batches with viability ≥80% were used.

Parasite tegumental antigens were extracted from PSC following original reports [27]. Briefly, 125,000 viable PSC per mL of extracting solution (MEGA-10 1% *w*/*v*, EDTA 5 mM and PMSF 2 mM in PBS) were incubated under mild constant rotation for 2 h at room temperature. Then, parasites were allowed to settle down and the supernatant was extensively dialyzed at room temperature against PBS using a cellulose membrane (MW cut-off: 12 KDa). Obtained soluble antigens (termed PSEx) were characterized in terms of protein content and stored at −20 °C until use [30].

Labeling of PSEx with fluorescein isothiocyanate (FITC) was performed as previously reported [14]. Briefly, 1 mg of PSEx was dialyzed against 100 mM NaHCO_3_ buffer pH 9, and then 500 μg of FITC (Sigma, St. Louis, MO, USA) dissolved in DMSO were added. After 8 h of vigorous shaking in the dark, the mixture was extensively dialyzed against PBS and finally stored at 4 °C until use.

### 4.3. PSEx Binding Assays

In vivo binding of PSEx to peritoneal exudate cells (PECs) was assessed after intraperitoneal (*i.p.*) infusion of PSEx-FITC to naïve mice. A group of C57Bl/6 WT mice (n = 7) received 100 μg/mouse of PSEx-FITC in sterile PBS (200 μL), while a control group (n = 3) received sterile PBS. At 30 min post-inoculation (*p.i.*), mice were euthanized, and PEC were individually collected through washouts using cold PBS supplemented with FCS 2% (*v*/*v*) [14]. Then, 2 × 10^5^ PEC from each mouse were stained with linage specific PE-conjugated antibodies (anti-CD19 for B cells: clone 1D3; anti-CD3 for T cells: clone 17A2; anti-CD49b for NK cells: clone DX5; or F4/80 for macrophages: clone BM8.1) in PBS with 0.5% BSA and 0.02% NaN_3_. All antibodies were from BD-Pharmingen, Franklin Lakes, NJ, USA.

Ex vivo binding of PSEx to B cells and macrophages from PEC were assessed using pooled PEC from WT, *Cd5*^−/−^ and *Cd6*^−/−^ C57BL/6 mice. PEC samples from naïve mice (n = 4/strain) were obtained as previously described and pooled accordingly. Then, 2 × 10^5^ PEC were incubated at 4 °C for 30 min in the presence of increasing amounts (1, 5, 15 and 25 μg) of PSEx-FITC or BSA-FITC, together with anti-CD19-PE or anti-F4/80-PE antibodies. Triplicates were at least analyzed.

Flow cytometry studies were performed using FACSCalibur^®^ equipment and CellQuest^®^ v3.1 software (Montevideo, Uruguay), or FACSCanto II^®^ equipment and FACSDiva^®^ v9.0 software (Barcelona, Spain) all from Becton Dickinson. Data were analyzed using Flowing Software 2.5.1 downloaded on https://bioscience.fi/cytometry-core/flowing-software (accessed on 1 March 2020).

### 4.4. PSEx Inoculation

*Cd5*^−/−^ and their WT littermate mice (n = 5/strain/treatment) were *i.p.* infused with either 50 μg/mouse of PSEx in sterile PBS (200 μL) or equal volume of sterile PBS. The same infusion procedure was applied to *Cd6*^−/−^ and their WT littermate mice. Peritoneal samples were obtained 48 h *p.i.* from each mouse following previous reports [41,59,74]. Briefly, individualized peritoneal cavities were first washed out using 1 mL/mouse of cold PBS supplemented with FCS 2% (*v*/*v*), and peritoneal cell-free exudates (finally stored at −20 °C for further antibody analysis) were obtained after centrifugation (1200 rpm for 7 min at 4 °C). Then, peritoneal washouts were repeated 4 times with 3 mL/mouse, lavages were centrifuged, and obtained PEC were pooled with the remaining pellets from the first washout. Erythrocytes were eliminated using Red Blood Cell Lysing Buffer^®^ (Sigma) following the manufacturer’s instructions. Finally, a third part of each PEC suspension was stored in TRIzol^®^ (Invitrogen, Carlsbad, CA, USA) at −80 °C for further qRT-PCR cytokine profiling, and the remaining cells were freshly used for flow cytometry analyses.

### 4.5. Cell Surface Activation Phenotyping

Activation of peritoneal B cells and macrophages from PSEx-treated mice was assessed by flow cytometry. PEC (2 × 10^5^) were FcR-blocked (mouse FcBlock, Becton Dickinson, Franklin Lakes, NJ, USA) and stained with the corresponding fluorochrome-conjugated antibodies from BD Pharmingen or Biolegend (San Diego, CA, USA): anti-CD80-FITC (clone 16-10A1), anti-CD86-FITC (clone GL1), anti-CD19-PE (clone 1D3) and/or anti-F4/80-PE (clone BM8.1). Data acquisition and flow cytometry analyses were performed as described above. To avoid potential mouse strain differences in basal expression levels, values were normalized to the corresponding median value of PBS-inoculated controls (either from KO or WT mice).

### 4.6. Cytokine Profiling

Cytokine profiling in PEC from PSEx-treated mice was characterized by RT-qPCR following previously reported protocols [59,81]. Briefly, RNA extraction was performed using TRIzol^®^ (Invitrogen) and DNA contamination was eliminated by DNasa I treatment (Invitrogen), both following manufacturer’s recommendations. Final RNA quality was controlled by means of usual determinations of absorbance 260-to-280 nm ratio. cDNA was synthesized by reverse transcription of 1 μg RNA using M-MLV-RT (Invitrogen) at 42 °C for 50 min. Mouse-specific primers (IFN-γ, TNF-α, IL-6, IL-12p40, IL-17A, IL-10, IL-4, IL-5, IL-13 and β-actin) were commercially obtained (Operon Biotechnologies Inc, Huntsville, AL, USA). qPCR reactions were performed using 0.9 μM of each specific primer and QuantiTect SYBRGreen PCR Kit^®^ (QIAGEN, Hilden, Germany) following the manufacturer’s instructions on a Rotor-Gene 6000 equipment (Corbett Life Science, Sydney, Australia). Cycling conditions were 95 °C for 15 min, 40 cycles at 95 °C for 15 s and 60 °C for 1 min, followed by a melting curve rising from 72 °C to 90 °C. β-actin was used as the normalizing gene and relative mRNA amounts were calculated using the 2^−ΔΔCt^ method [89]. Levels of cytokine transcripts (mRNA) were normalized to the mean value from the corresponding PBS-treated controls (either from KO or WT mice).

### 4.7. Natural Polyreactive Antibodies

Natural polyreactive antibodies (NAb) in peritoneal cell-free exudates from PSEx-treated mice were characterized using DNP (2,4-dinitrophenol) as a surrogating antigen [90], following previous reports [26,40]. Briefly, 96-well microtiter plates were coated with DNP-BSA (5 μg/mL) diluted in PBS, and then blocked using PBS containing BSA 1% (*w*/*v*). Samples were diluted in PBS containing BSA 0.5% (*w*/*v*) plus Tween-20 0.05% (*v*/*v*), and incubated overnight at 4 °C. After washing, appropriate dilutions of peroxidase-conjugated antibodies specific to mouse IgM, IgA, IgG1, IgG2c, IgG2b or IgG3 were incubated for 2 h at 37 °C. Finally, peroxidase activity was detected using H_2_O_2_ (1% *v*/*v*) and TMB (6 mg/mL) in sodium acetate 100 mM pH 5.5 buffer. The absorbance was measured at 450 nm. Levels of NAb were defined as the absorbance value obtained at an all-samples non-saturating dilution for each isotype/subclass analyzed. To avoid potential mouse strain differences in basal NAb levels, values were normalized to the corresponding median value of PBS-inoculated controls (either from KO or WT mice).

### 4.8. Statistics

Data processing was performed using Microsoft Office Excel^®^, while graphs and statistical analyses were performed with GraphPad Prism^®^ v10.x. Group outliers identified by the ROUT method (Q = 1%) were routinely removed before statistical analyses. Parametric data (e.g., analytical replicates) were compared using Student’s *t*-test or one-way ANOVA depending on the comparisons performed, while for non-parametric data (e.g., biological replicates) Mann–Whitney–Wilcoxon test was applied. Differences were always regarded as significant at *p* < 0.05.

## 5. Conclusions

In summary, the results presented here showed that SR-I CD5 and CD6 receptors played direct and indirect roles in the early recognition of tegumental antigens from *E. granulosus* s.l. by peritoneal immune cells. Consequently, deficient CD5 and CD6 expression led to remarkable functional consequences on PEC activation, antibody secretion and cytokine production; all corresponding to immune parameters previously suggested to influence the host resistance to CE. Although further and deeper studies are warranted for a detailed dissection of CD5 and CD6 functional roles during helminth infections, relevant evidence was herein obtained to highlight their importance in the complex host–parasite interplay.

## Data Availability

The original contributions presented in this study are included in the article/Supplementary Materials. Further inquiries can be directed to the corresponding authors.

## Supplementary Materials

The following supporting information can be downloaded at https://www.mdpi.com/article/10.3390/ijms27062870/s1.
