# Supplementary material for: The Class I Scavenger Receptors CD5 and CD6 Play a Role in the Early Peritoneal Immune Response to Echinococcus granulosus Tegumental Antigens
Source: Int J Mol Sci. 2026 Mar 22;27(6):2870. doi: 10.3390/ijms27062870 (PMC13026194; doi:10.3390/ijms27062870)
Supplement: Supplementary file 1 [file ijms-27-02870-s001.zip › ijms-4196061-supplementary.pdf]

Supplementary Material

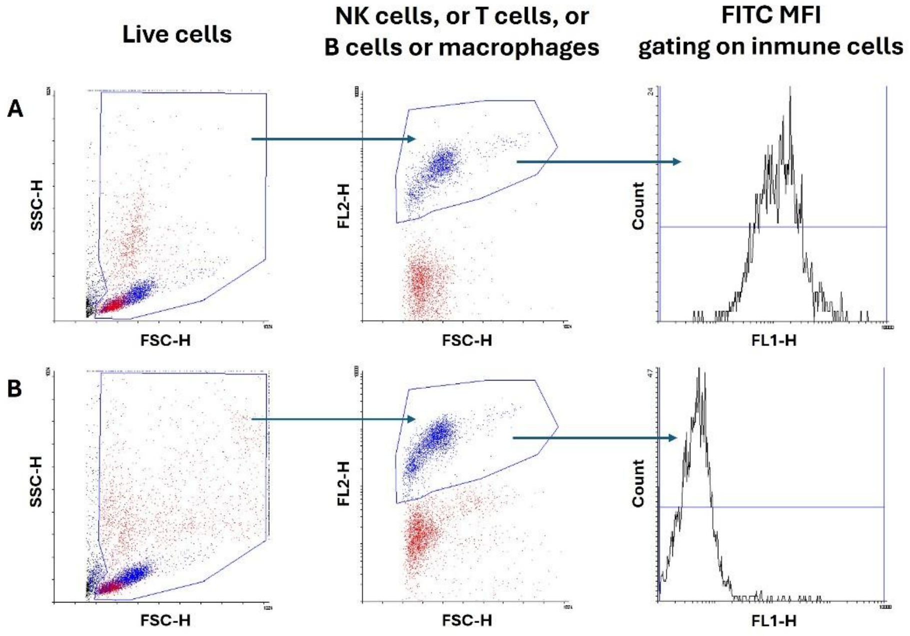

Figure S1. Gating strategy for the in vivo assessment of PSEx binding to PEC. In vivo interaction between PEC and PSEx was determined by flow cytometry in PEC isolated from C57Bl/6 mice ip inoculated with PSEx-FITC (100 µg) or PBS. PEC were recovered 30 min *p.i.*, and were incubated with lineage-specific PE-conjugated antibodies (NK cells: CD49b^{+}, T cells: CD3^{+}, B cells: CD19^{+} and macrophages: F4/80^{+} cells). General gating strategies for PEC from PSEx- **(A)** or PBS-inoculated **(B)** mice are shown.

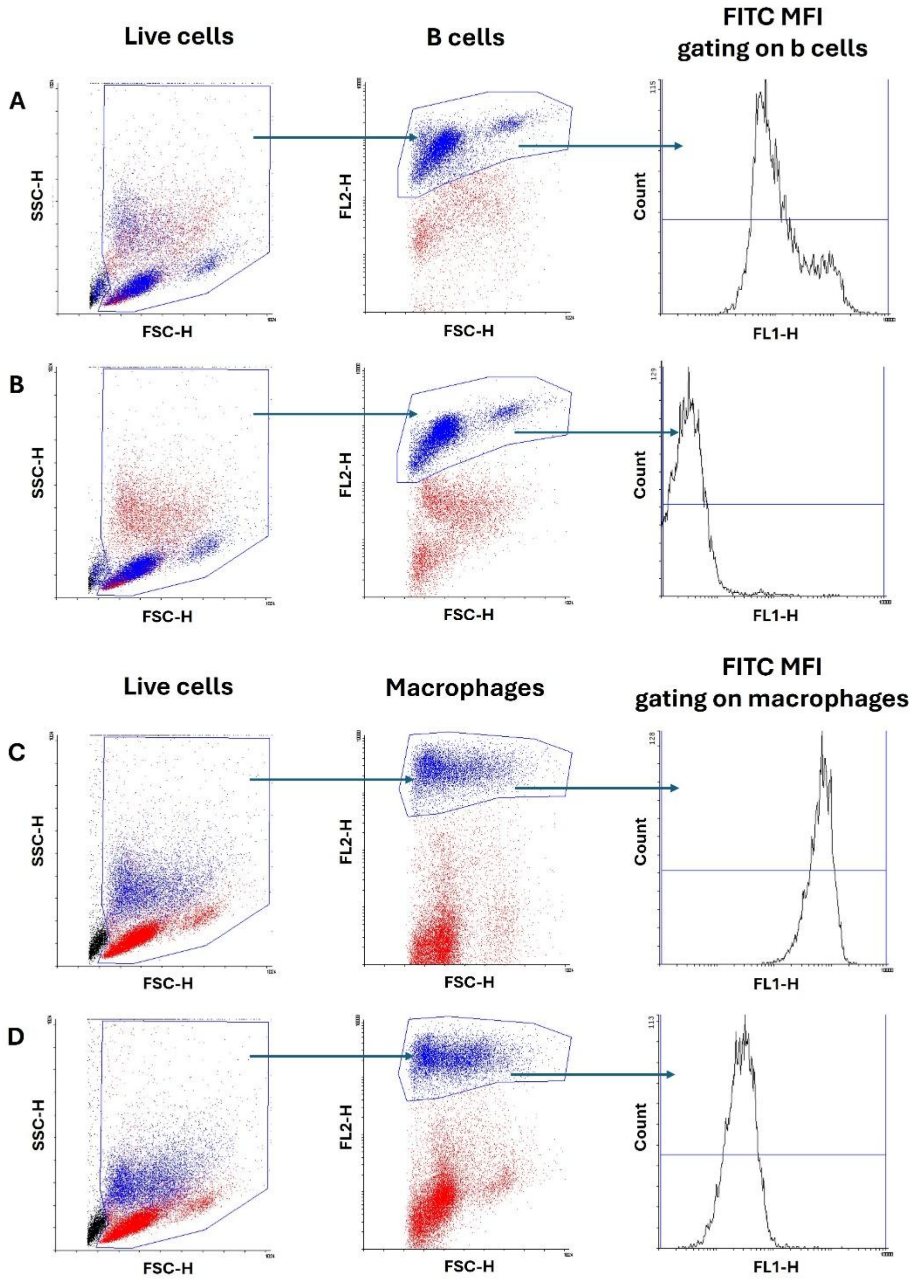

Figure S2. Gating strategy for the in vitro characterization of PSEx binding to B cells and macrophages. In vitro interaction between peritoneal B cells **(A, B)** and macrophages **(C, D)** with PSEx determined by flow cytometry. PEC isolated from naïve C57Bl/6 mice were incubated with increasing amounts (1, 5, 15 and 25 μg) of PSEx-FITC **(A, C)** or BSA-FITC **(B, D)**, and then labeled with anti-CD19 **(A, B)** or anti-F4/80 **(C, D)** PE-conjugated antibodies.
